# An appraisal of respiratory system compliance in mechanically ventilated covid-19 patients

**DOI:** 10.1186/s13054-021-03518-4

**Published:** 2021-06-09

**Authors:** Gianluigi Li Bassi, Jacky Y. Suen, Heidi J. Dalton, Nicole White, Sally Shrapnel, Jonathon P. Fanning, Benoit Liquet, Samuel Hinton, Aapeli Vuorinen, Gareth Booth, Jonathan E. Millar, Simon Forsyth, Mauro Panigada, John Laffey, Daniel Brodie, Eddy Fan, Antoni Torres, Davide Chiumello, Amanda Corley, Alyaa Elhazmi, Carol Hodgson, Shingo Ichiba, Carlos Luna, Srinivas Murthy, Alistair Nichol, Pauline Yeung Ng, Mark Ogino, Antonio Pesenti, Huynh Trung Trieu, John F. Fraser, the COVID-19 Critical Care Consortium, Tala Al-Dabbous, Huda Alfoudri, Mohammed Shamsah, Subbarao Elapavaluru, Ashley Berg, Christina Horn, Stephan Schroll, Jorge Velazco, Wanda Fikes, Ludmyla Ploskanych, Dan Meyer, Maysoon Shalabi-McGuire, Trent Witt, Ashley Ehlers, Lorenzo Grazioli, E. Wilson Grandin, Jose Nunez, Tiago Reyes, Mark Joseph, Brook Mitchell, Martha Tenzer, Ryuzo Abe, Yosuke Hayashi, Hwa Jin Cho, In Seok Jeong, Nicolas Brozzi, Jaime Hernandez-Montfort, Omar Mehkri, Stuart Houltham, Jerónimo Graf, Rodrigo Perez, Roderigo Diaz, Camila Delgado, Joyce González, Maria Soledad Sanchez, Diego Fernando Bautista Rincón, Melissa Bustamante Duque, Angela Maria Marulanda Yanten, Dan Brodie, Desy Rusmawatiningtyas, Gabrielle Ragazzo, Azhari Taufik, Margaretha Gunawan, Vera Irawany, Muhammad Rayhan, Elizabeth Yasmin Wardoyo, Mauro Panigada, Silvia Coppola, Sebastiano Colombo, Giacomo Grasselli, Michela Leone, Alberto Zanella, Massimo Antonelli, Simone Carelli, Domenico L. Grieco, Motohiro Asaki, Kota Hoshino, Leonardo Salazar, Laura Duarte, Joseph McCaffrey, Allison Bone, David Thomson, Christel Arnold-Day, Jerome Cupido, Zainap Fanie, Malcom Miller, Lisa Seymore, Dawid van Straaten, Ibrahim Hassan, Ali Ait Hssain, Jeffrey Aliudin, Al-Reem Alqahtani, Khoulod Mohamed, Ahmed Mohamed, Darwin Tan, Joy Villanueva, Ahmed Zaqout, Ethan Kurtzman, Arben Ademi, Ana Dobrita, Khadija El Aoudi, Juliet Segura, Gezy Giwangkancana, Shinichiro Ohshimo, Koji Hoshino, Saito Hitoshi, Yuka Uchinami, Javier Osatnik, Anne Joosten, Antoni Torres, Ana Motos, Minlan Yang, Carlos Luna, Francisco Arancibia, Virginie Williams, Alexandre Noel, Nestor Luque, Trieu Huynh Trung, Sophie Yacoub, Marina Fantini, Ruth Noemi Jorge García, Enrique Chicote Alvarez, Anna Greti, Oscar Lomeli, Adrian Ceccato, Angel Sanchez, Ana Loza Vazquez, Ferran Roche-Campo, Divina Tuazon, Toni Duculan, Hiroaki Shimizu, Marcelo Amato, Luciana Cassimiro, Flavio Pola, Francis Ribeiro, Guilherme Fonseca, Heidi Dalton, Mehul Desai, Erik Osborn, Hala Deeb, Antonio Arcadipane, Claudia Bianco, Raffaele Cuffaro, Gennaro Martucci, Giovanna Occhipinti, Matteo Rossetti, Chiara Vitiello, Sung-Min Cho, Kate Calligy, Glenn Whitman, Hiroaki Shimizu, Naoki Moriyama, Jae-Burm Kim, Nobuya Kitamura, Takashi Shimazui, Abdullah Al-Hudaib, Alyaa Elhazmi, Johannes Gebauer, Toshiki Yokoyama, Abdulrahman Al-Fares, Esam Alamad, Fatma Alawadhi, Kalthoum Alawadi, Sarah Buabbas, Hiro Tanaka, Satoru Hashimoto, Masaki Yamazaki, Tak-Hyuck Oh, Mark Epler, Cathleen Forney, Jared Feister, Katherine Grobengieser, Louise Kruse, Joelle Williamson, Eric Gnall, Mara Caroline, Sasha Golden, Colleen Karaj, Sherry McDermott, Lynn Sher, Timothy Shapiro, Lisa Thome, Mark Vanderland, Mary Welch, Luca Brazzi, Tawnya Ogston, Dave Nagpal, Karlee Fischer, Roberto Lorusso, Maria de Piero, Mariano Esperatti, Diarmuid O’Briain, Edmund G. Carton, Ayan Sen, Amanda Palacios, Deborah Rainey, Cassandra Seefeldt, Lucia Durham, Octavio Falcucci, Amanda Emmrich, Jennifer Guy, Carling Johns, Emily Neumann, Nina Buchtele, Michael Schwameis, Stephanie-Susanne Stecher, Delila Singh, Michaela Barnikel, Lukas Arenz, Akram Zaaqoq, Lan Anh Galloway, Caitlin Merley, Marc Csete, Luisa Quesada, Isabela Saba, Daisuke Kasugai, Hiroaki Hiraiwa, Taku Tanaka, Eva Marwali, Yoel Purnama, Santi Rahayu Dewayanti, Ardiyan, Debby Siagian, Yih-Sharng Chen, John Laffey, Bairbre McNicholas, David Cosgrave, Marlice VanDyk, Sarah MacDonald, Ian Seppelt, Indrek Ratsep, Lauri Enneveer, Kristo Erikson, Getter Oigus, Andra-Maris Post, Piret Sillaots, Frank Manetta, Mamoru Komats, S. Veena Satyapriya, Amar Bhatt, Marco Echeverria, Juan Fiorda, Alicia Gonzalez, Nahush A. Mokadam, Johnny McKeown, Joshua Pasek, Haixia Shi, Alberto Uribe, Rita Moreno, Bishoy Zakhary, Hannah Johnson, Nolan Pow, Marco Cavana, Alberto Cucino, Giuseppe Foti, Marco Giani, Vincenzo Russotto, Davide Chiumello, Valentina Castagna, Andrea Dell’Amore, Hoi-Ping Shum, Alain Vuysteke, Asad Usman, Andrew Acker, Blake Mergler, Nicolas Rizer, Federico Sertic, Benjamin Smood, Alexandra Sperry, Madhu Subramanian, Navy Lolong, Ernita Akmal, Erlina Burhan, Menaldi Rasmin, Bhat Naivedh, Faya Sitompu, Peter Barrett, Julia Daugherty, David Dean, Antonio Loforte, Irfan Khan, Olivia DeSantis, Mohammed Abraar Quraishi, Gavin Salt, Dominic So, Darshana Kandamby, Jose M. Mandei, Hans Natanael, Eka YudhaLantang, Anastasia Lantang, Anna Jung, Terese Hammond, George Ng, Wing Yiu Ng, Pauline Yeung, Shingo Adachi, Pablo Blanco, Ana Prieto, Jesús Sánchez, Meghan Nicholson, Michael Farquharson, Warwick Butt, Alyssa Serratore, Carmel Delzoppo, Pierre Janin, Elizabeth Yarad, Richard Totaro, Jennifer Coles, Robert Balk, Samuel Fox, James Hays, Esha Kapania, Pavel Mishin, Andy Vissing, Garrett Yantosh, Saptadi Yuliarto, Kohar Hari Santoso, Susanthy Djajalaksana, Arie Zainul Fatoni, Masahiro Fukuda, Keibun Liu, Paolo Pelosi, Denise Battaglini, Juan Fernando Masa Jiménez, Sérgio Gaião, Roberto Roncon-Albuquerque, Jessica Buchner, Young-Jae Cho, Sang Min Lee, Su Hwan Lee, Tatsuya Kawasaki, Pranya Sakiyalak, Prompak Nitayavardhana, Tamara Seitz, Rakesh Arora, David Kent, Swapnil Parwar, Andrew Cheng, Jennene Miller, Daniel Marino, Jillian E. Deacon, Shigeki Fujitani, Naoki Shimizu, Jai Madhok, Clark Owyang, Hergen Buscher, Claire Reynolds, Olavi Maasikas, Aleksandr Beljantsev, Vladislav Mihnovits, Takako Akimoto, Mariko Aizawa, Kanako Horibe, Ryota Onodera, Carol Hodgson, Meredith Young, Timothy Smith, Cheryl Bartone, Timothy George, Kiran Shekar, Niki McGuinness, Lacey Irvine, Brigid Flynn, Abigail Houchin, Keiki Shimizu, Jun Hamaguchi, Leslie Lussier, Grace Kersker, John Adam Reich, Gösta Lotz, Maximilian Malfertheiner, Esther Dreier, Lars Maier, Neurinda Permata Kusumastuti, Colin McCloskey, Al-Awwab Dabaliz, Tarek B. Elshazly, Josiah Smith, Konstanty S. Szuldrzynski, Piotr Bielański, Yusuff Hakeem, Keith Wille, Rebecca Holt, Ken Kuljit S. Parhar, Kirsten M. Fiest, Cassidy Codan, Anmol Shahid, Mohamed Fayed, Timothy Evans, Rebekah Garcia, Ashley Gutierrez, Hiroaki Shimizu, Tae Song, Rebecca Rose, Suzanne Bennett, Denise Richardson, Giles Peek, Dalia Lopez-Colon, Lovkesh Arora, Kristina Rappapport, Kristina Rudolph, Zita Sibenaller, Lori Stout, Alicia Walter, Daniel Herr, Nazli Vedadi, Lace Sindt, Cale Ewald, Julie Hoffman, Sean Rajnic, Shaun Thompson, Ryan Kennedy, Matthew Griffee, Anna Ciullo, Yuri Kida, Ricard Ferrer Roca, Cynthia Alegre, Sofia Contreras, JordI Riera, Christy Kay, Irene Fischer, Elizabeth Renner, Hayato Taniguci, Gabriella Abbate, Halah Hassan, Silver Heinsar, Varun A. Karnik, Katrina Ki, Hollier F. O’Neill, Nchafatso Obonyo, Leticia Pretti Pimenta, Janice D. Reid, Kei Sato, Kiran Shekar, Aapeli Vuorinen, Karin S. Wildi, Emily S. Wood, Stephanie Yerkovich

**Affiliations:** 1 Critical Care Research Group, The Prince Charles Hospital, Chermside, Australia; 2 University of Queensland, Brisbane, Australia; 3 Institut d’Investigacions Biomèdiques August Pi i Sunyer, Barcelona, Spain; 4 Queensland University of Technology, Brisbane, Australia; 5 St Andrew’s War Memorial Hospital, UnitingCare Hospitals, Brisbane, Australia; 6 Wesley Medical Research, Brisbane, Australia; 7 INOVA Fairfax Medical Center, Heart and Vascular Institute, Falls Church, VA USA; 8 University of Pau et Pays De L’Adour, LMAP, E2S-UPPA, CNRS, Pau, France; 9 Macquarie University, Sydney, Australia; 10 Roslin Institute, University of Edinburgh, Edinburgh, UK; 11 Queen Elizabeth II University Hospital, Glasgow, UK; 12 Fondazione IRCCS Ca’ Granda Ospedale Maggiore Policlinico di Milano, Milan, Italy; 13 Anaesthesia and Intensive Care Medicine, National University of Ireland, Galway, Ireland; 14 Department of Medicine, Columbia College of Physicians and Surgeons, and Center for Acute Respiratory Failure, New-York-Presbyterian Hospital, New York, NY USA; 15 Interdepartmental Division of Critical Care Medicine, University of Toronto, Toronto, Canada; 16 Department of Medicine, University of Toronto, Toronto, Canada; 17 Institute of Health Policy, Management and Evaluation, University of Toronto, Toronto, Canada; 18 Toronto General Hospital Research Institute, University of Toronto, Toronto, Canada; 19 Hospital Clinic of Barcelona, Barcelona, Spain; 20 Ospedale San Paolo, Milan, Italy; 21 University of Milan, Milan, Italy; 22 King Faisal Specialist Hospital and Research Centre, Riyadh, Saudi Arabia; 23 The Alfred Hospital, Melbourne, Australia; 24 Australian and New Zealand Intensive Care Research Centre, Department of Epidemiology and Preventive Medicine, School of Public Health, Monash University, Melbourne, Australia; 25 University College Dublin-Clinical Research Centre at St Vincent’s University Hospital, Dublin, Ireland; 26 Intensive Care, Nippon Medical School Hospital, Tokyo, Japan; 27 Neumonología, Hospital de Clínicas, UBA, Buenos Aires, Argentina; 28 Department of Pediatrics, Faculty of Medicine, University of British Columbia, Vancouver, Canada; 29 The University of Hong Kong, Hong Kong, China; 30 Nemours Alfred I duPont Hospital for Children, Wilmington, DE USA; 31 Hospital for Tropical Diseases, Ho Chi Minh City, Vietnam; 32 Australian Centre for Health Services Innovation (AusHSI) and Centre for Healthcare Transformation, School of Public Health and Social Work, Queensland University of Technology (QUT), Brisbane, QLD Australia

**Keywords:** Mechanical ventilation, Compliance, ARDS, COVID-19, SARS-CoV-2

## Abstract

**Background:**

Heterogeneous respiratory system static compliance (*C*_RS_) values and levels of hypoxemia in patients with novel coronavirus disease (COVID-19) requiring mechanical ventilation have been reported in previous small-case series or studies conducted at a national level.

**Methods:**

We designed a retrospective observational cohort study with rapid data gathering from the international COVID-19 Critical Care Consortium study to comprehensively describe *C*_RS_—calculated as: tidal volume/[airway plateau pressure-positive end-expiratory pressure (PEEP)]—and its association with ventilatory management and outcomes of COVID-19 patients on mechanical ventilation (MV), admitted to intensive care units (ICU) worldwide.

**Results:**

We studied 745 patients from 22 countries, who required admission to the ICU and MV from January 14 to December 31, 2020, and presented at least one value of *C*_RS_ within the first seven days of MV. Median (IQR) age was 62 (52–71), patients were predominantly males (68%) and from Europe/North and South America (88%). *C*_RS_, within 48 h from endotracheal intubation, was available in 649 patients and was neither associated with the duration from onset of symptoms to commencement of MV (*p* = 0.417) nor with PaO_2_/FiO_2_ (*p* = 0.100). Females presented lower *C*_RS_ than males (95% CI of *C*_RS_ difference between females-males: − 11.8 to − 7.4 mL/cmH_2_O *p* < 0.001), and although females presented higher body mass index (BMI), association of BMI with *C*_RS_ was marginal (*p* = 0.139). Ventilatory management varied across *C*_RS_ range, resulting in a significant association between *C*_RS_ and driving pressure (estimated decrease − 0.31 cmH_2_O/L per mL/cmH_2_0 of *C*_RS_, 95% CI − 0.48 to − 0.14, *p* < 0.001). Overall, 28-day ICU mortality, accounting for the competing risk of being discharged within the period, was 35.6% (SE 1.7). Cox proportional hazard analysis demonstrated that *C*_RS_ (+ 10 mL/cm H_2_O) was only associated with being discharge from the ICU within 28 days (HR 1.14, 95% CI 1.02–1.28, *p* = 0.018).

**Conclusions:**

This multicentre report provides a comprehensive account of *C*_RS_ in COVID-19 patients on MV. *C*_RS_ measured within 48 h from commencement of MV has marginal predictive value for 28-day mortality, but was associated with being discharged from ICU within the same period. Trial documentation: Available at https://www.covid-critical.com/study.

*Trial registration*: ACTRN12620000421932.

## Background

Millions of people have been infected by SARS-CoV-2 worldwide, and many of those have been hospitalized for respiratory complications associated with coronavirus disease-2019 (COVID-19). Many of those COVID-19 hospitalised patients have received mechanical ventilation (MV), due to the development of acute hypoxemic respiratory failure and acute respiratory distress syndrome (ARDS) [1–4]. To date, several landmark studies [5–8] have improved our understanding of COVID-19 pulmonary pathophysiology, but pulmonary derangement in COVID-19 and appropriate ventilatory management remains incompletely characterized.

Earlier reports on the pulmonary pathophysiology of COVID-19 patients reported conflicting results and extreme heterogeneity in levels of pulmonary shunting, static respiratory system compliance (*C*_RS_), [9–12] and substantial heterogeneity in lung recruitability [13, 14]. Adding further to the controversy over *C*_RS_ in COVID-19 patients, Grasselli and collaborators [7] have compared findings from an Italian repository of COVID-19 ARDS with previous ARDS cases of different etiologies. They found statistically significant higher *C*_RS_ in patients with COVID-19 ARDS. In addition, they found that patients who presented with lower *C*_RS_ and higher D-dimer values had the greatest mortality risk. In line with these figures, in a small-case series, Chiumello and collaborators found that COVID-19 patients presented higher *C*_RS_ levels in comparison with patients with ARDS from other etiologies and matched levels of hypoxemia [12]. Regrettably, those previous reports did not provide any information on how *C*_RS_ progressed beyond a punctual assessment during the period of MV. In contrast, in another landmark study by Ferrando et al. [6], *C*_RS_ figures from a Spanish database were very similar to previously published cohorts of ARDS patients. The authors also found that intensive care unit (ICU) discharge and mortality were not influenced by the initial levels of *C*_RS_.

In a pandemic caused by a novel virus, access to international data is vital, because it may help account for differences in populations, access to medical care, equipment and critical variations in clinical managements among countries. Thus, analysis of international repositories improves the overall understanding of a novel disease and helps establishing best practices to enhance outcome. One example of how single-center or single-country studies can influence medical care early in a pandemic, before being contradicted by subsequent international findings is the issue of *C*_RS_. Indeed, as this parameter can be markedly impacted by fine variations in ventilatory management, extrapolations from mono-center or single-country studies may be challenging. In early January 2020, the COVID-19 Critical Care Consortium incorporating the ExtraCorporeal Membrane Oxygenation for 2019 novel Coronavirus Acute Respiratory Disease (COVID-19–CCC/ECMOCARD) group was founded to investigate patients presenting to ICUs worldwide.

Here, we present a comprehensive appraisal of *C*_RS_ in mechanically ventilated COVID-19 patients enrolled into the COVID-19–CCC/ECMOCARD international study, in order to understand the dynamics of *C*_RS_ during the first week of mechanical ventilation and its potential impact on patient outcomes.

## Materials and methods

### Study design and oversight

The COVID-19-CCC/ECMOCARD is an international, multicentre, cohort observational study ongoing in 351 hospitals across 53 countries. The full study protocol is available elsewhere [15]. To summarize, participating hospitals obtained local ethics committee approval and a waiver of informed consent was granted in all cases. ISARIC/SPRINT-SARI data collection began at admission to hospital, while data collection for the COVID-19–CCC observational study commenced at admission to the ICU. De-identified patient data were collected retrospectively and stored via the REDCap electronic data capture tool, hosted at the University of Oxford, United Kingdom or Monash University, Melbourne, Australia.

### Study population

We reviewed data of all patients admitted to the ICU at a COVID-19–CCC collaborating site, from January 14 through September 30, 2020, with a clinically suspected or laboratory confirmed diagnosis of SARS-CoV-2 infection, through naso-pharyngeal swab for real-time PCR SARS-CoV-2 detection. Of note, suspicion of SARS-CoV-2 infection was based on symptoms and onset of infection and was confirmed by the clinician when COVID-19 infection was the most likely cause of the symptoms experienced. Patients excluded were those under the age of 15 years or admitted to an ICU for other reasons. We focused our analysis on patients on controlled MV and with a computed *C*_RS_ value within 48 h of MV commencement.

### Definitions and pulmonary mechanics computations

*C*_RS_ was calculated as: tidal volume (mL)/[(airway plateau pressure-PEEP (cmH_2_O))]. Of note, we provided to data collectors a detailed data dictionary, with instructions on how to collect airway plateau pressure values, via an inspiratory pause of approximately 3 s. We computed *C*_RS_ using the first measured tidal volume, airway plateau pressure and PEEP values, within 48 h of MV commencement. In the sub-population of patients on controlled MV, without ECMO support, we analysed key pulmonary variables, such as tidal volume, positive end expiratory pressure (PEEP), static driving pressure, inspiratory fraction of oxygen (FiO_2_), and gas exchange, recorded during routine clinical practice and only. Tidal volume was reported in mL/kg of predicted body weight (PBW) [16].

### Data collection

After enrolment, data on demographics, comorbidities, clinical symptoms and laboratory results were collected by clinical and research staff of the participating ICUs in an electronic case report form [15]. Details of respiratory and hemodynamic support, physiological variables, and laboratory results were collected daily. Of note, the worst daily values were preferentially recorded. The duration of MV and ICU stay, and hospital mortality were recorded. Analysis of daily data was restricted to the first seven days from commencement of MV.

### Statistical analyses

Descriptive statistics summarised demographics, clinical signs on ICU admission, ICU management and clinical outcomes for the overall study cohort and subjects with baseline compliance measured within the first 48 h of controlled MV. Statistics were reported as medians (interquartile range) for continuous variables and numbers (percentage) for categorical variables. Linear regression was applied to summarise associations between baseline compliance with body mass index (BMI) (including interaction between BMI and sex), days from symptom onset to MV commencement and PaO_2_/FiO_2_, adjusted for BMI. Linear mixed modelling was used to investigate trends in compliance over time and associations with key respiratory parameters during the first 7 days of controlled MV. Models assumed a linear effect for days and a random intercept per subject to account for repeated measures. Consistent with exploratory analyses, BMI was included as a fixed effect to adjust for potential confounding in the clinical characteristics and management of patients with different BMI. Hypothesis testing was applied to all fixed effects, assuming a 5% level of statistical significance. Results were summarised graphically with uncertainty in estimated trends represented by 95% prediction intervals. Expected patient outcomes including length of ICU stay, duration of MV and risk of ICU mortality versus discharge were examined using multi-state modelling [17]. Compared with exploratory analyses of clinical outcomes, the multistate model accounted for ICU discharge and death as competing events and allowed data from all patients to be included, regardless of study follow-up time. The model comprised of four states, to describe patients prior to commencement of MV (non MV), on mechanical ventilation (MV), ICU discharged (Discharge) and mortality (Death). States were presented as percentage and standard error (SE) in the text. Patients extubated before death or discharge were assumed to transition between MV an non-MV states. State transitions were modelled by Cox proportional hazards, with patients censored at last known follow-up, up to 28 days from ICU admission. Follow-up analysis considered Cox proportional hazard regression to examine associations between baseline compliance and competing risks of ICU mortality and discharge, following commencement of MV. Baseline compliance was included as a linear effect, with age, sex, BMI and comorbidities (hypertension, chronic cardiac disease, chronic kidney disease) as additional covariates and adjusted for recruiting centre. A shared frailty term (Gamma distributed) was included to account for residual variation between study sites. Analyses were conducted using R version 3.6.2 or higher (The R Foundation).

## Results

**Fig. 1:**
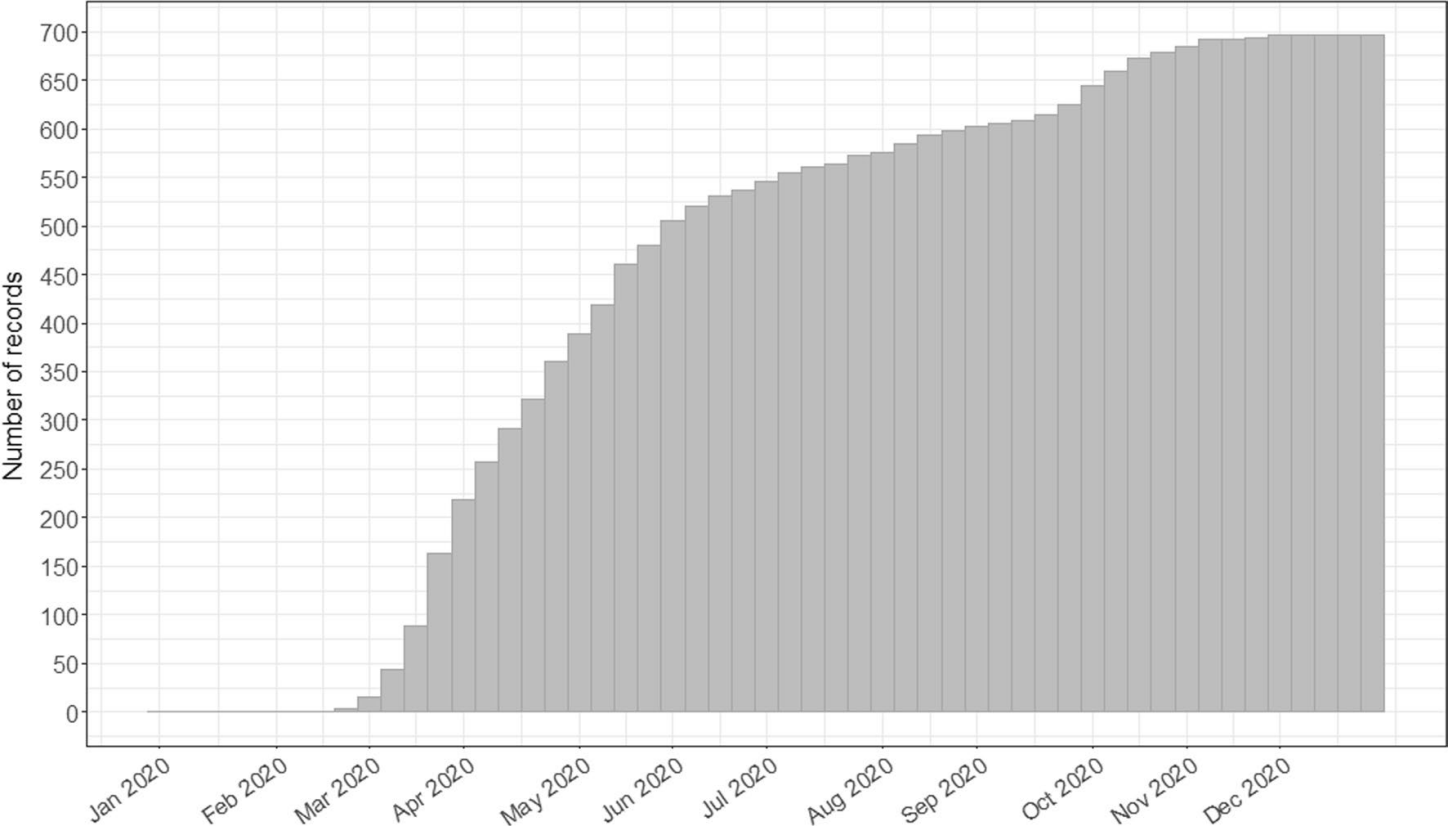
Patient enrolment rate from January 14 through December 31, 2020

**Fig. 2:**
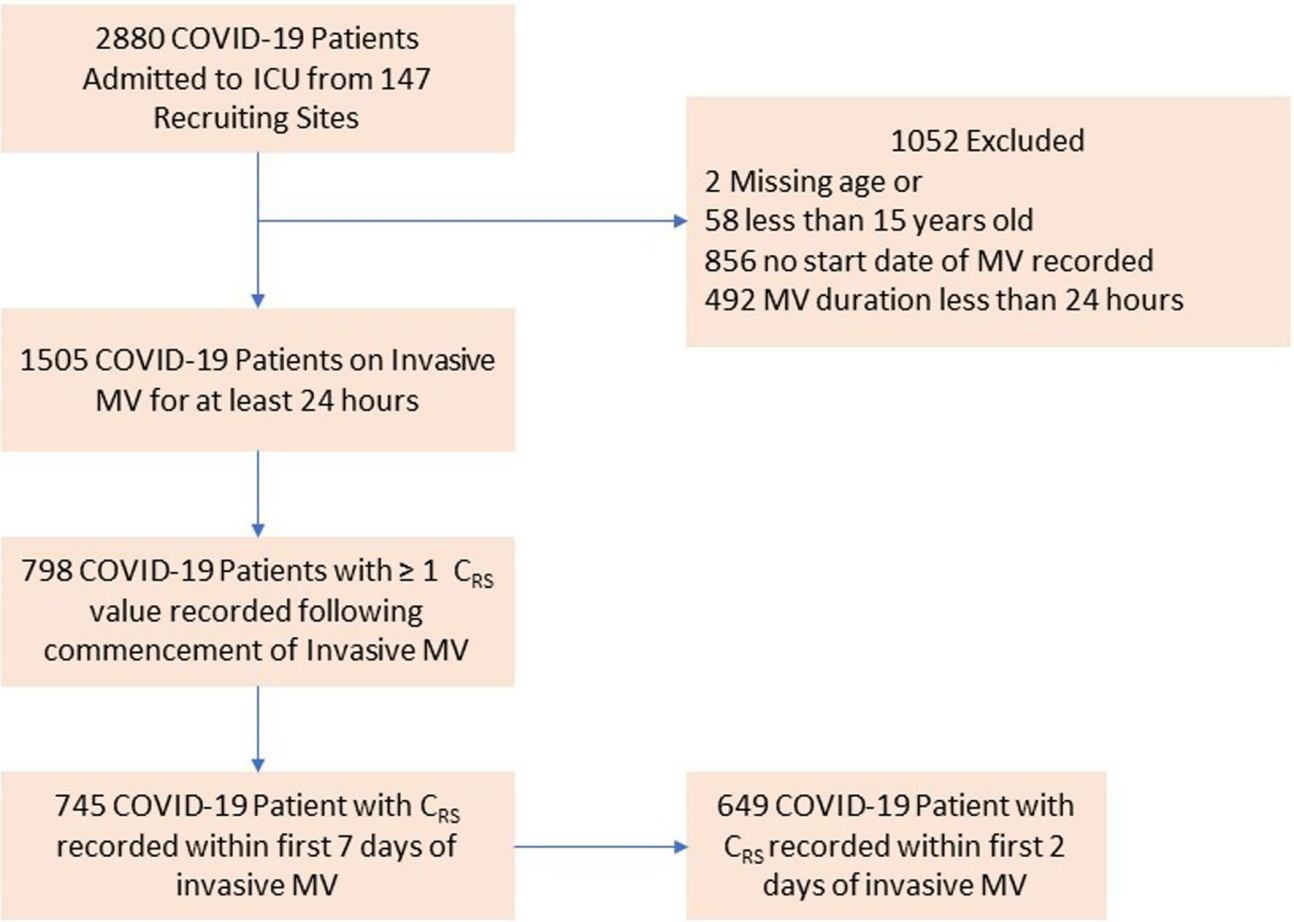
Patient population flow chart. The analysis of 1505 COVID-19 patients on mechanical ventilation identified 649 patients with static respiratory system compliance within 48 h from commencement of mechanical ventilation

**Fig. 3:**
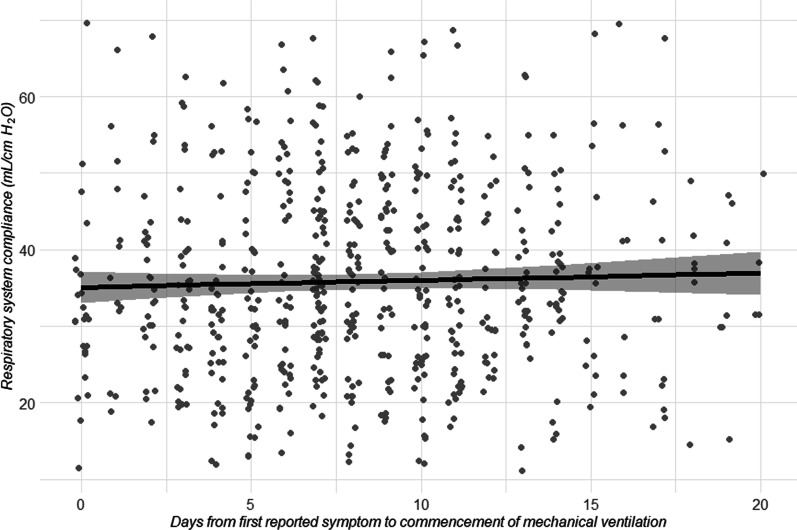
Linear regression analysis of days from onset of symptoms to commencement of mechanical ventilation and static respiratory system compliance, based on the first measurement obtained within 48 h from commencement of mechanical ventilation, adjusted for body mass index. Dark black horizontal bar depicts median value, and upper and lower horizontal light black bars show 90th and 10th percentile. Days of onset of symptoms to commencement of mechanical ventilation was not associated with static respiratory system compliance (estimate 0.92 mL/cmH_2_O, 95% CI − 0.31–0.31 *p* = 0.417)

**Fig. 4:**
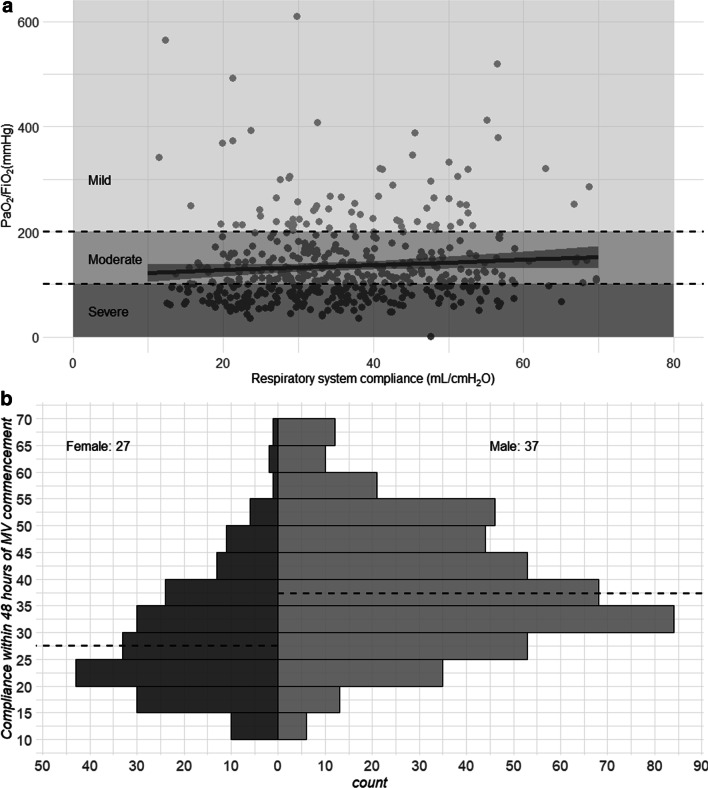
**a** Linear regression analysis of arterial partial pressure of oxygen (PaO_2_/FiO_2_) and respiratory system compliance (*C*_RS_), based on the first measurement obtained within 48 h from commencement of mechanical ventilation, with an interaction of gender and adjusted for body mass index (BMI). No statistically significant association was found between PaO_2_/FiO_2_ and *C*_RS_ (estimate 0.49, 95% CI − 0.09–1.07 *p* = 0.100). Typical acute respiratory distress syndrome stratification groups [35] (severe, moderate and mild based on levels of hypoxemia) are highlighted in dark, medium and light grey, respectively. **b** Static respiratory system compliance (*C*_RS_) distribution by sex, based on the first measurement obtained within 48 h from commencement of mechanical ventilation. Dashed black lines depict median values for females and males

**Fig. 5:**
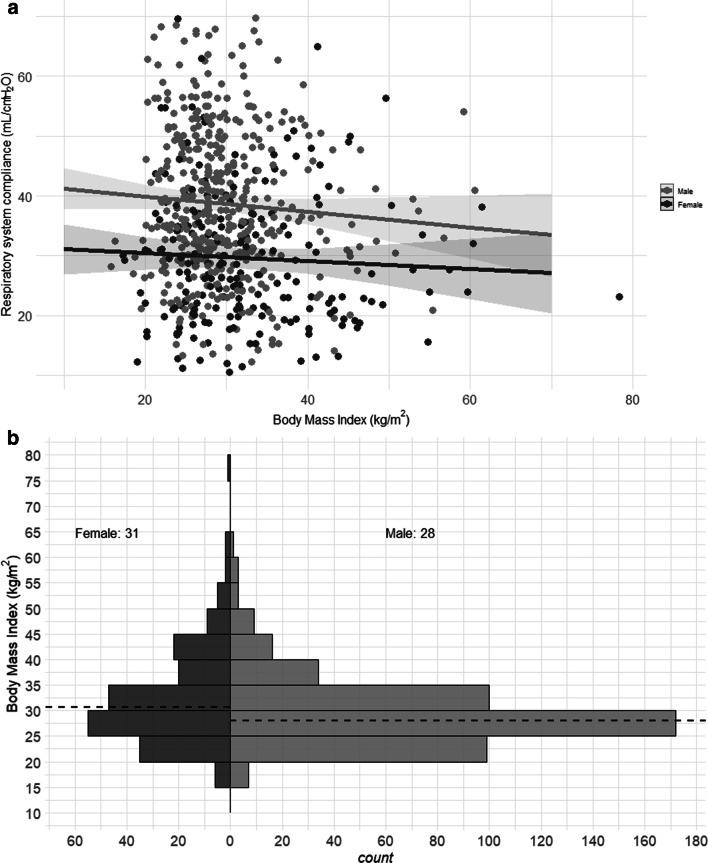
Linear regression analysis of static respiratory system compliance, based on the first measurement obtained within 48 h from commencement of mechanical ventilation, and body mass index with an interaction for sex. Per each graph, fitted line of the model is depicted and the upper and lower lines display the 95% predictive interval. Dark grey dots depict female patients, while light grey dots males. Static respiratory system compliance did not vary according to the body mass index (estimate − 0.12 cmH_2_O/mL, 95%CI − 0.29 to − 0.04, *p* = 0.139), but was associated with female sex (estimate − 10.73 cmH_2_O/mL, 95%CI − 18.54 to − 2.92, *p* = 0.007)

**Fig. 6:**
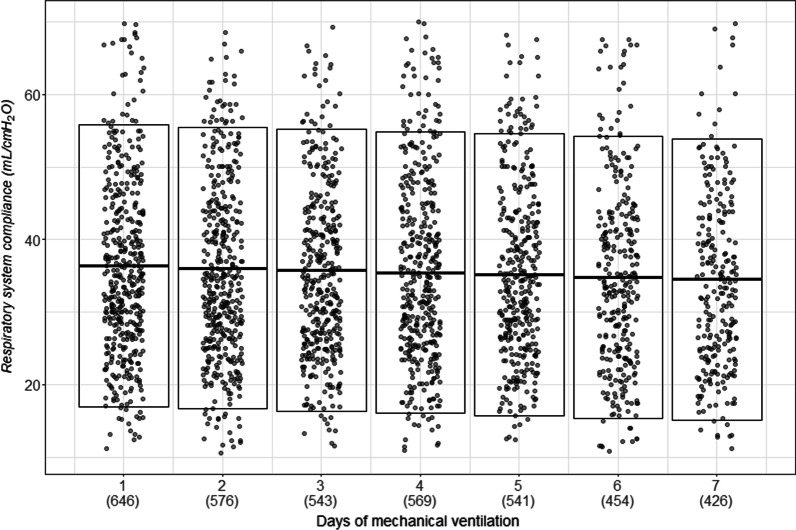
Static respiratory system compliance dynamics. Evolution of static respiratory system compliance over the first 7 days of mechanical ventilation, adjusted for body mass index. Under each day, the number of analysed patients is reported in parenthesis. Fitted line of the model is depicted, and the upper and lower lines display the 95% predictive interval. Respiratory system compliance varied during the first seven days of mechanical ventilation (estimate − 0.31 cmH_2_O/mL, 95%CI − 0.48 to 0.14, *p* < 0.001)

**Fig. 7:**
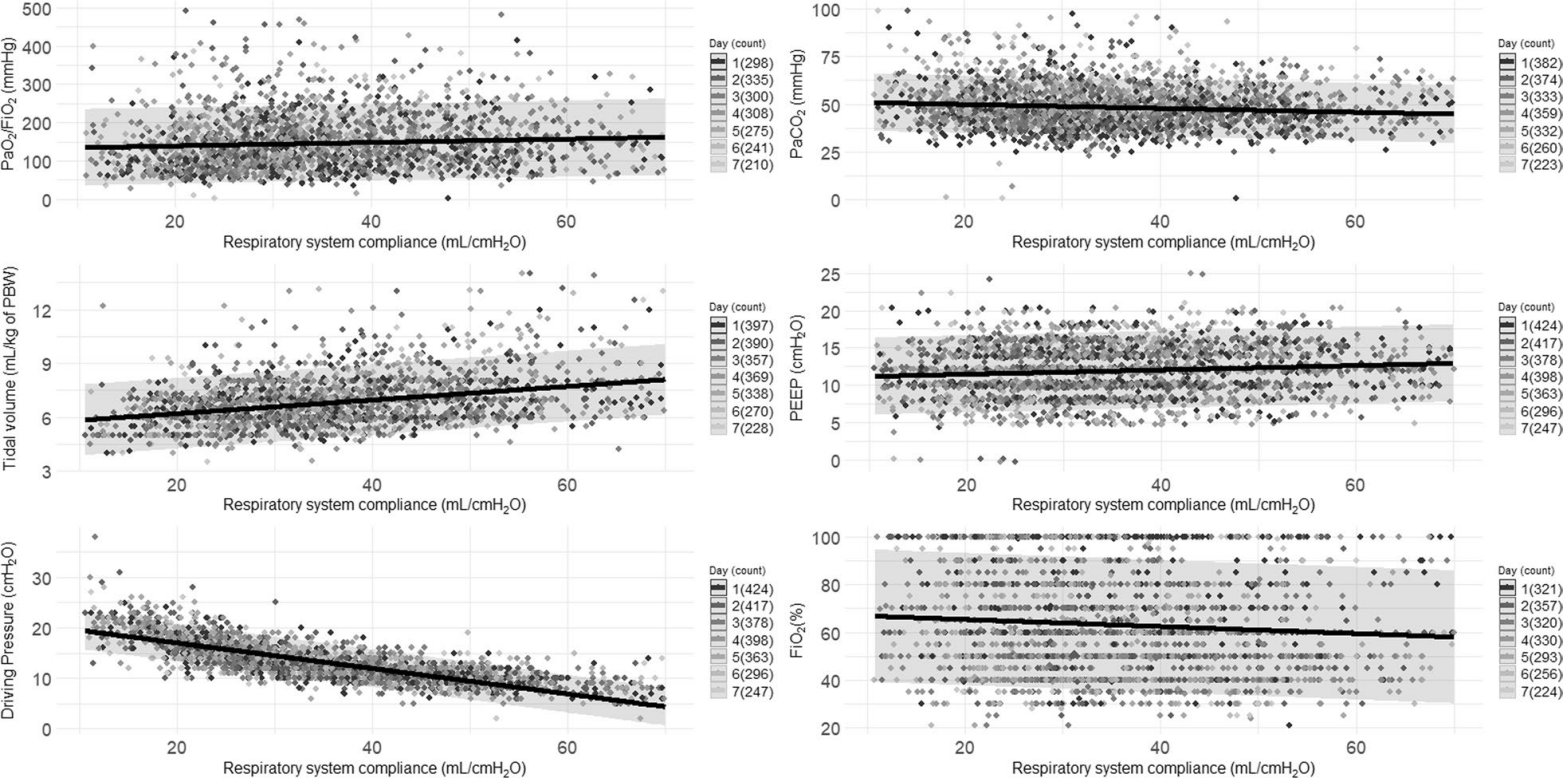
Linear Mixed model analysis of respiratory system compliance vs. crucial pulmonary variables during the first 7 days of mechanical ventilation (grey-scale coded bar for day 1 through 7 is reported on the right section of each graph and in parenthesis is reported the number of analysed patients). Per each graph, fitted line of the model is depicted and the upper and lower lines display the 95% predictive interval. All analyses are adjusted for body mass index. Static compliance of respiratory system was found to be associated with PaCO_2_ (estimated decrease − 0.11 mmHg, 95% CI − 0.15 to − 0.06, *p* < 0.001), tidal volume (estimated increase 0.04 mL/Kg of predicted body weight per day, 95% CI 0.03–0.04, *p* < 0.001), PEEP (estimated increase − 0.03 cmH_2_O, 95% CI 0.02–0.04, *p* < 0.001), driving pressure (estimated decrease − 0.31 cmH_2_O/L, 95% CI − 0.48 to 0.14, *p* < 0.001) and FiO_2_ (estimated decrease − 0.15%, 95% CI − 0.23 to − 0.06, *p* < 0.001). While PaO_2_/FiO_2_, was not significantly associated with static compliance of respiratory system (estimated increase 0.29 mmHg, 95% CI − 0.03 to 0.61, *p* = 072) PaO_2_/FiO_2,_ ratio between arterial partial pressure of oxygen and inspiratory fraction of oxygen; PaCO_2_ arterial partial pressure of carbon dioxide; PEEP, positive end-expiratory pressure

**Fig. 8:**
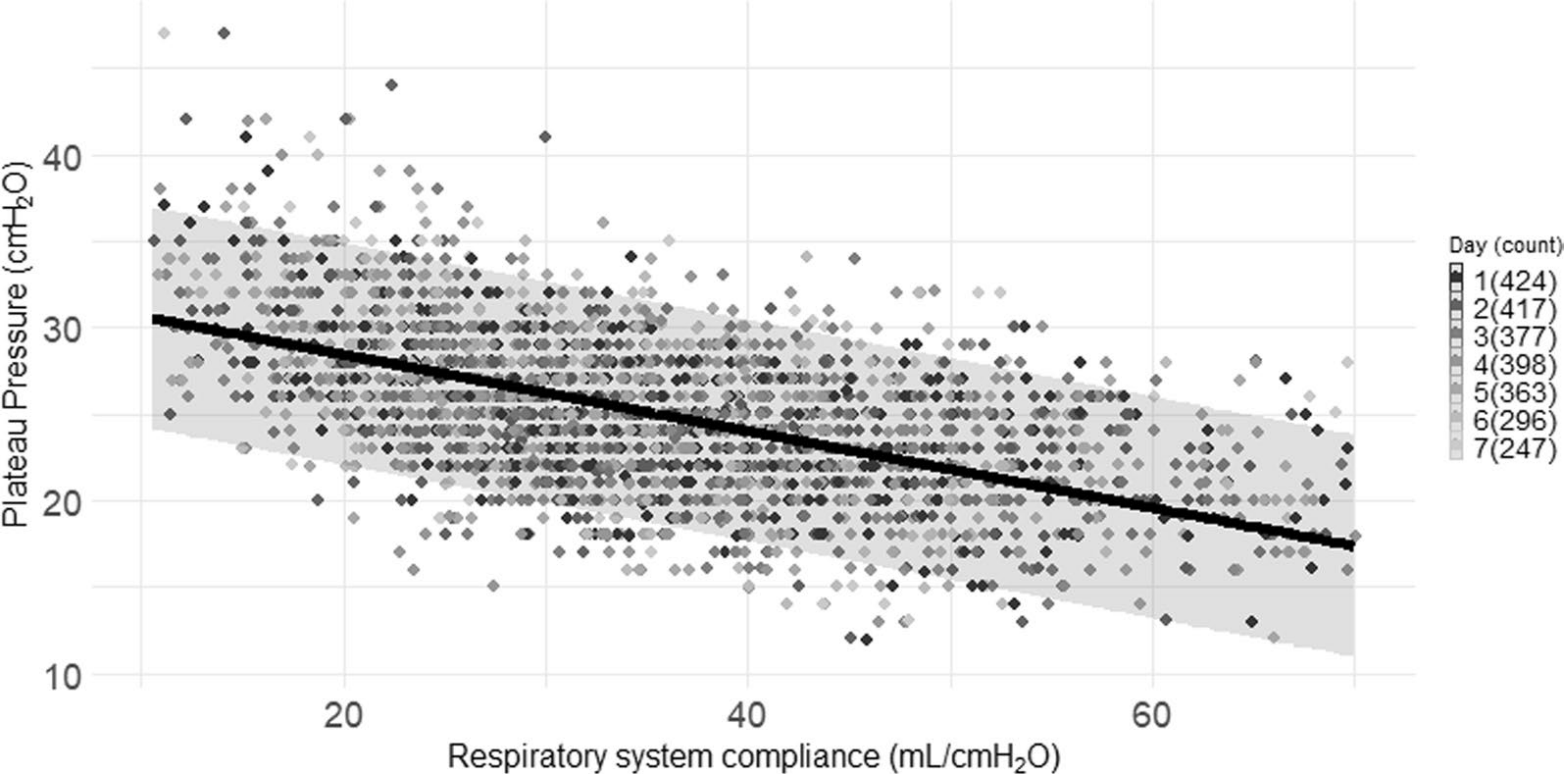
Association of airway inspiratory plateau pressure with static respiratory system compliance. Linear Mixed model analysis of the association of respiratory system compliance with airway inspiratory plateau pressure during the first 7 days of mechanical ventilation (grey-scale coded bar for day 1 through 7 is reported on the right section of each graph and in parenthesis is reported the number of analysed patients). Fitted line of the model is depicted, and the upper and lower lines display the 95% predictive interval. Analysis is adjusted for body mass index. The model highlights significant association between respiratory system compliance and airway plateau pressure (estimated decrease − 0.22 cmH_2_O/L, 95% CI − 0.23 to − 0.21, *p* < 0.001), but based on the model prediction, airway plateau pressure remained predominantly below 30 cmH_2_O

We studied 745 patients from 22 countries, who required admission to the ICU and MV from January 14 to December 31, 2020, and presented at least one value of *C*_RS_ within the first seven days of MV. Among those, 597 (80%) had laboratory-confirmed diagnosis of SARS-CoV2 infection, while in 148 (20%), infection was clinically suspected. Enrolment rate, since January 2020, is reported in Fig. 1. *C*_RS_, within 48 h from endotracheal intubation, was available in 649 patients (Fig. 2). No association between *C*_RS_ and days from onset of symptoms to commencement of MV was found (Fig. 3). Median *C*_RS_ (IQR), within the first 48 h of mechanical ventilation, was 34.1 mL/cmH_2_O (26.4–44.0) and PaO_2_/FiO_2_ 113.0 mmHg (84.0–161.3), without any linear association between these parameters. In particular, 16%, 46% and 38% of the patients presented with mild, moderate or severe hypoxemia, respectively (Fig. 4a). Female sex was associated with a significantly lower *C*_RS_ than in males (95% CI of difference between genders: − 11.8 to − 7.4 mL/cmH_2_O *p* < 0.001) (Fig. 4b). Females also presented higher body mass index (BMI) (95% CI of difference between males and females: − 1.9 to − 5.5, *p* < 0.001), but as shown in Fig. 5, *C*_RS_ and BMI were not linearly associated. Our model estimated that *C*_RS_ was 37.57 cmH_2_O/mL (95% CI 36.5–38.6) upon commencement of MV (Fig. 6), with further worsening in the first seven days of MV (estimated decrease − 0.31 cmH_2_O/mL per day, 95% CI − 0.48 to − 0.14, *p* < 0.001). In addition, as detailed in Fig. 7, PaCO_2_, tidal volume, PEEP, driving pressure and FiO_2_ significantly varied across the range of *C*_RS_, and a significant association was found between inspiratory plateau pressure and *C*_RS_ changes (Fig. 8).

**Fig. 9:**
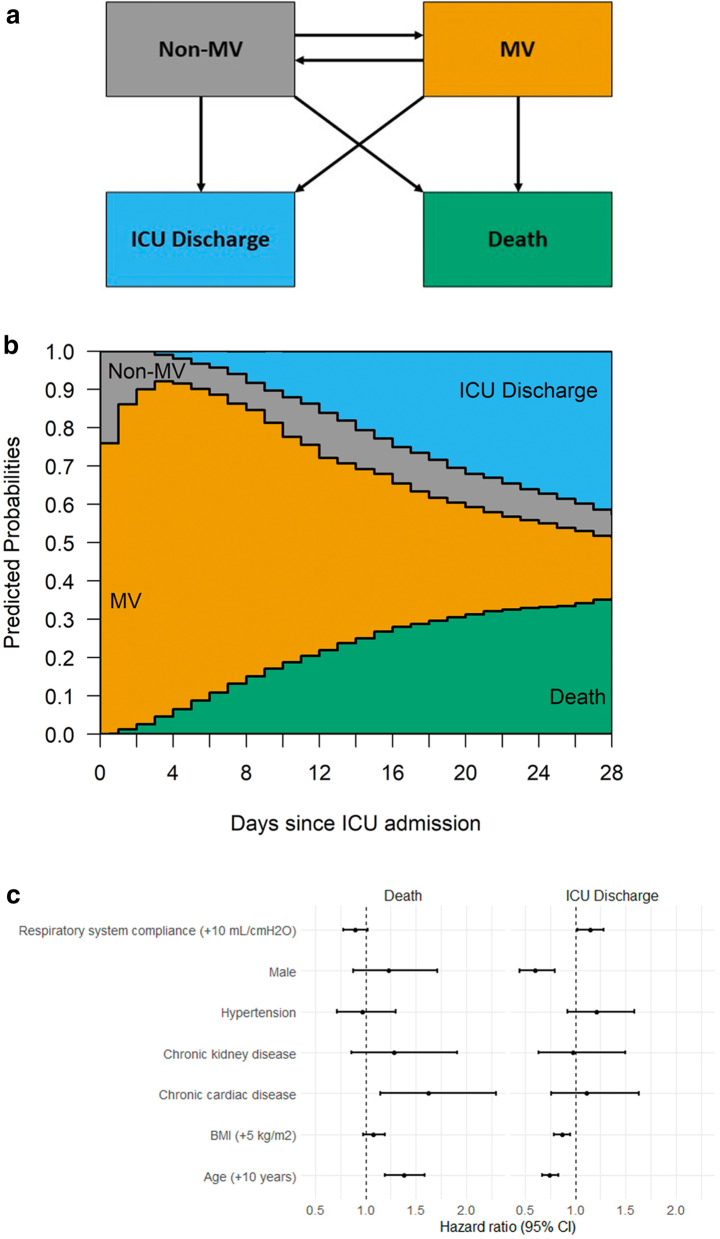
Multistate modelling and Cox regression analysis outcomes for patient with static compliance recorded within 48 h of commencing mechanical ventilation. **a** Multistate model structure for estimating expected outcomes up to 28 days from admission to intensive care unit (ICU). Modelled health states include not on invasive mechanical ventilation (non-MV), on mechanical ventilation (MV), ICU discharge and death. Patients start in the non-MV state if not mechanically ventilated upon or prior to ICU admission, or in the MV state otherwise. **b** Predicted probabilities of occupying health states up to 28 days from ICU admission. **c** Results of Cox proportional hazards modelling for risk of death and ICU discharge from commencement of mechanical ventilation. Covariates comprise age, body mass index (BMI), selected comorbidities (hypertension, chronic cardiac disease, chronic kidney disease) and baseline static compliance. Parameter estimates are presented as estimated hazard ratios with 95% confidence intervals (CI). Further details on factors significantly associated with assessed outcomes are available in the results section

Baseline characteristics upon ICU admission, applied interventions and outcomes, are summarized in Table 1. The most common interventions applied to the study population were use of antibiotics (96%), neuromuscular blocking agents (81%) and prone position (61%). The overall hospital mortality of the study population was 40%, and among those patients who died in the hospital or were discharged alive, the median (IQR) duration of MV was 11 days (6–18) and 14 days (8–23), respectively. Overall, 28-day ICU mortality, accounting for competing risks, was 35.6% (SE 1.7) and estimated 28-day mortality from commencement of MV was 37.1% (SE 1.7) (Fig. 9b). Cox proportional hazard analysis (Fig. 9c) demonstrated that age (hazard ratio 1.37, 95% CI 1.19–1.59, *p* < 0.001) and chronic cardiac diseases (HR 1.62, 95% CI 1.14–2.29, *p* < 0.001) were the only baseline factors associated with 28-day mortality risk. In addition, age (HR 0.77, 95% CI 0.66–0.83, *p* < 0.001), male sex (HR 0.59, 95% CI 0.44–0.79, *p* < 0.001), BMI (HR 0.86, 95% CI 0.79–0.95, *p* = 0.003) and *C*_RS_ (+ 10 mL/cm H_2_O) (HR 1.14, 95% CI 1.02–1.28, *p* = 0.018) were associated with the chance of being discharge from the ICU within 28 days.

**Table 1:** Only patients with the following characteristics were included in this analysis: (1) on controlled mechanical ventilation; (2) airway plateau pressure, tidal volume and positive-end-expiratory pressure recorded within 48 h from commencement of mechanical ventilation

Characteristic	Full cohort (*n* = 745)	First *C*_RS_ recorded within 48 hr of MV (*n* = 649)
Age, years: *n*; median (IQR)	745; 62 (52–71)	649; 62 (53–71)
Male: *n* (%)	510 (68)	445 (69)
Geographic region: *n* (%)		
Africa	19 (3)	14 (2)
Asia	63 (8)	57 (9)
Australia and New Zealand	6 (1)	4 (1)
Europe	326 (44)	295 (45)
Latin America and the Caribbean	108 (14)	92 (14)
Northern America	223 (30)	187 (29)
Time from onset of symptoms, days: *n*; median (IQR)		
Onset of symptoms to hospital admission	735; 7 (3–9)	643; 7 (3–9)
Onset of symptoms to ICU admission	735; 7 (5–11)	643; 7 (5–11)
Onset symptoms to mechanical ventilation	735; 8 (5–11)	643; 8 (5–11)
Clinical signs on ICU admission: *n*; median (IQR)		
WBC count, 10^3^/µL	604; 8.9 (6.3–12.8)	540; 9.0 (6.6–13.0)
Lymphocyte count, 10^3^	459; 0.7 (0.5–1.1)	402; 0.7 (0.5–1.1)
Temperature, °C	329; 37.4 (36.5–38.1)	293; 37.4 (36.5–38.2)
Creatinine, mg/dL	613; 1.0 (0.7–1.4)	543; 1.0 (0.7–1.4)
CRP, mg/dL	216; 118.1 (29.4–206.7)	194;121 (29.1–205.6)
Lymphocyte count to CRP ratio	167; 0.01 (0–0.03)	147; 0.01 (0–0.03)
Neutrophil to Lymphocyte ratio	414; 10.1 (5.6–16.6)	364;10.2 (5.9–16.6)
D-dimer level mg/L	237; 1.3 (0.8–4.7)	207; 1.4 (0.8–4.4)
Clinical management during first 28 days of ICU admission: *n* (%)		
Antibiotics	713 (96)	621 (96)
Antivirals	288 (50)	245 (49)
Continuous renal replacement therapy	110 (15)	92 (15)
Vasoactive drugs	411 (58)	365 (58)
Cardiac-assist devices	54 (7)	48 (7)
ECMO	72 (10)	61 (9)
Prone positioning	451 (61)	392 (60)
Inhaled nitric oxide	72 (10)	66 (10)
Neuromuscular blockade^a^	599 (81)	524 (81)
Recruitment manoeuvres	295 (40)	266 (41)
Clinical outcomes		
Outcome at study end: *n* (%)		
Died in hospital	300 (40)	266 (41)
Discharged alive	400 (54)	339 (52)
Transferred to another facility	7 (1)	7 (1)
Still in hospital/outcome not finalised	38 (5)	37 (6)
Died in hospital		
Duration of ICU stay, days: *n*; median (IQR)	300; 12 (6–20)	266; 12 (6–20)
Duration of hospital stay, days: *n*, Median (IQR)	294; 13 (7–22)	260; 14 (7–22)
Duration of MV, days: *n*; Median (IQR)	300; 11 (6–18)	266; 11 (5–18)
Died within 28 days from ICU admission: *n* (%)	258 (86)	231 (87)
Discharged alive		
Duration of ICU stay, days: *n*; median (IQR)	399; 19 (12–30)	339; 19 (11–3)
Duration of hospital stay, days: *n*; Median (IQR)	396; 30 (21–46)	336; 30 (21–45)
Duration of MV, days: *n*; Median (IQR)	400; 14 (8–23)	339; 14 (8–23)
Discharged alive within 28 days from ICU admission: *n* (%)	195 (49)	165 (49)

Percentages are calculated for non-missing data

*C*_RS_, static compliance of respiratory system; CRP, c-reactive protein; MV, mechanical ventilation; ICU, intensive care unit; IQR, interquartile range; ECMO, extracorporeal membrane oxygenation

^a^Administration of neuromuscular blockade drugs administered during the first day of invasive mechanical ventilation was not included in the analysis

## Discussion

This large observational report from intensive care units throughout the world found that initial static respiratory system compliance was only associated with hazard of being discharged from the ICU within 28 days. The duration from onset of symptoms to commencement of MV did not influence *C*_RS_, and interestingly lower *C*_RS_ was found in female patients. In the evaluated population, neuromuscular blocking agents and prone position were commonly applied and ventilatory management across *C*_RS_ levels varied in terms of tidal volume, PEEP and FiO_2_, throughout the first 7 days of MV.

In comparison with previous reports on ARDS patients without COVID-19 [18], we similarly found that the majority of patients exhibited moderate hypoxemia, even when presented higher *C*_RS_. We also noted a larger range of *C*_RS_ in line with previous studies [7, 8], but in contrast with values from a larger COVID-19 ARDS series from Spain [6]. Considering that we focused our analysis on static compliance of the respiratory system, without partitioning into the pulmonary and chest wall components [19, 20], it is interesting that *C*_RS_ was not associated with BMI, suggesting that patients with higher BMI potentially presented also with higher lung compliance. Irrespective, we found lower *C*_RS_ in female patients, who also presented higher BMIs. To the best of our knowledge, no studies have systematically investigated the effects of gender/BMI on COVID-19 severity; thus, whether obesity might be a crucial risk factor for ICU admission and mechanical ventilation, specifically in female patients, and its effects on lung compliance should be further explored. We also found that throughout the range of *C*_RS_ values, plateau pressure was within what is typically presumed as lung protective ranges [21], but this resulted in potentially harmful driving pressures, specifically for patients with the lowest *C*_RS_ values. As many of these patients were obese, this raises the question of whether these modest pressures might have increased the risk of pulmonary derecruitment, or in patients with normal BMI, the resulting driving pressure might have been related to pulmonary overdistention. These factors could have contributed to sustained hypoxemia and impaired lung function throughout the study period. In such circumstances, it is questionable whether MV guided by oesophageal pressure monitoring may have some benefits [22], but more research is needed to corroborate such reasoning.

Phenotypic subsets of COVID-19-associated ARDS have been proposed [9, 13, 23–25]. Recent study has also explored whether *C*_RS_—related phenotype patterns existed among patients with ARDS before the COVID-19 pandemic [26]. Various investigators [7, 27], who did not find significant *C*_RS_ variability among COVID-19 patients requiring MV, questioned the overall clinical value of *C*_RS_ in the COVID-19 population. In a very small case series, Gattinoni et al [9] found an initial *C*_RS_ of 50 mL/cmH_2_O, but high levels of shunt fraction that could have explained the resulting severe hypoxemia. In subsequent study, Chiumello and collaborators found higher *C*_RS_ in patient with COVID-19 ARDS and ARDS caused by other injuries, while matching for similar levels of PaO_2_/FiO_2_ [12]. Interestingly, these findings were in line with computed tomography studies results, corroborating higher proportion of normally aerated tissue in COVID-19 ARDS. In similar reports, heterogeneous pathophysiology among patients with different levels of pulmonary compliance has been implied [10, 25]. As corroborated by landmark post-mortem studies [28] and clinical studies [7, 29], SARS-CoV-2 heterogeneously affects pulmonary ventilation and perfusion. Hence, it could be argued that the use of *C*_RS_ as key pathophysiological parameter to predict clinical evolution might be over simplistic and in-depth characterization of pulmonary pathophysiology should be recommended for COVID-19 patients, specifically when obese. Interestingly, our report is the first that specifically focused on the dynamics of *C*_RS_, rather than only baseline *C*_RS_. We found that *C*_RS_ was not related to the duration from the onset of symptoms to commencement of MV, emphasising the need for inclusive data on mechanisms of lung injury in not ventilated COVID-19 patients [30]. The median *C*_RS_ value found in our population was 34.1 mL/cmH_2_O, similar to findings by Ferrando et al. [6], not dissimilar to findings by Bellani et al. on patients with non-COVID-19 ARDS [31], but lower than figures recently reported by Grasselli [7] and Grieco [32] in COVID-19 patients. In addition, we found a further decrease in *C*_RS_ during the first week of MV. This could have been related to the specific ventilatory management in our reported population, but such discrepancy further highlights the need of a comprehensive appraisal of pulmonary and chest wall mechanics in COVID-19 patients [20].

One of the most striking results was the continued use of high PEEP over the first seven days of MV, even in patients with high compliance. This seems counterintuitive, given that current recommendations in ARDS suggest decreasing PEEP, especially in the face of high compliance. As hypoxemia persisted even with high PEEP and high compliance, our results add to the hypothesis that maintaining high PEEP may worsen gas exchange from lung overdistension, resulting in increased dead space and intrapulmonary shunting. Other authors have speculated that using high levels of PEEP in COVID-19 patients with low recruitability may be detrimental, and that lowering PEEP may improve gas exchange and limit ventilator-induced lung injury [33]. Our results in this large cohort of patients from multiple global areas support this theory. Finally, we found that patients required two weeks of MV, and 28-day mortality in the overall population was 35.6%, with hospital mortality up to 40%. These figures are in line with mortality rates reported by Grasselli [7] in the subgroups characterized by low D-dimer, and mortality in severe-moderate COVID-19 ARDS, as corroborated by Ferrando [6]. Nevertheless, we found that *C*_RS_ was only associated with the discharge from ICU within 28 days. Thus, the marginal clinical value of *C*_RS_ as a predictor of mortality in COVID-19 patients calls for urgent identification of valuable markers that could inclusively describe pulmonary derangement and guide personalized treatment.

### Strengths and limitations

Collaborations between international data collection efforts have the ability to answer many questions related to COVID 19 and to pave the way for future novel diseases to achieve rapid and global data access to help guide best practice. The international COVID-19 Critical Care Consortium study [15], in collaboration with the ISARIC/SPRINT-SARI networks [34], provides inferences not limited by ventilatory management specific to small patient cohort or single-country studies. In addition, in comparison with previous studies, we provided more granular data to inclusively appraise the dynamics of *C*_RS_ in COVID-19 patients on MV and to study its association with laboratory, and clinical features. A few limitations of our observational study should also be emphasized. First, we centred our analysis on COVID-19 patients, without comparisons against previous repositories of patients with ARDS from different aetiologies. Yet, we provided a wide-ranging discussion of the characteristics of our population in the context of previous analyses in ARDS patients. Second, inferences on pulmonary perfusion disorders in our population can only be speculative, since D-dimer was only available in a small subset of patients (Table 1). Third, as reported by the enrolment rate (Fig. 1 Supplemental Digital Content), patients were mostly enrolled in the early phase of the pandemic, hence extrapolations from our findings should take into account potential biases related to overwhelmed critical care services. Fourthly, it is important to emphasise that we centred our analysis on *C*_RS_, but due to the complex respiratory pathophysiology in COVID-19 patients and the high percentage of patients with increased BMI, the use of oesophageal pressure monitoring to fully describe lung and chest wall compliances is advisable and should be prioritised in future investigations. Fifth, the majority of patients were admitted in centers located in North America, Europe and South America. Although these findings are in line with the global distribution of COVID-19 cases, extrapolations of our findings in other regions should be applied cautiously.

### Conclusions

Our comprehensive appraisal of COVID-19 patients on MV from a large international observational study implies that expected *C*_RS_ within 48 h from commencement of MV is not influenced by the duration from onset of symptoms to commencement of MV, but after intubation, a further decrease in *C*_RS_ might be expected during the first week of ventilation. In addition, baseline *C*_RS_ is associated with the chance of being discharged from the ICU within 28 days, but it is not a predictive marker of 28-day mortality. Based on potential inferences from our findings, future studies that could provide an in-depth characterization of lungs and chest wall compliance in COVID-19 patients will be critical to guide best practice in ventilatory management.

## Data Availability

The datasets used and/or analysed during the current study are available from the corresponding author on reasonable request.

## Abbreviations

ARDS: Acute respiratory distress syndrome
COVID-19: Coronavirus disease-2019
COVID-19–CCC/ECMOCARD: COVID-19 Critical Care Consortium incorporating the ExtraCorporeal Membrane Oxygenation for 2019 novel Coronavirus Acute Respiratory Disease
FiO_2_: Inspiratory fraction of oxygen
ICU: Intensive care unit
IQR: Interquartile range
MV: Mechanical ventilation
PBW: Predicted body weight
PEEP: Positive end expiratory pressure
*C*_RS_: Static respiratory system compliance

## Declarations

### Statistical analysis

Nicole White; Sally Shrapnel; Benoit Liquet; Samuel Hinton; Aapeli Vuorinem; Gareth Booth.
